# Review: Impact of food safety on global trade

**DOI:** 10.1002/vms3.1585

**Published:** 2024-08-19

**Authors:** Abebe Tibebu, Habtamu Tamrat, Adane Bahiru

**Affiliations:** ^1^ Sekota Dryland Agricultural Research Center Sekota Ethiopia; ^2^ College of Agriculture and Environmental Sciences Bahir Dar University Bahir Dar Ethiopia

**Keywords:** foodborne diseases, food safety, international trade, Public Health and World Trade Organization

## Abstract

Food safety encompasses the supply and assurance of safe, high‐quality food for consumers. It is a crucial aspect of food security, gaining greater global attention due to the increasing number of widespread foodborne incidents. International trade is expanding as countries increasingly rely on each other to secure a sufficient and diverse food supply. Beyond this, concerns about food safety have become more prevalent due to various factors. Therefore, this review aims to investigate the effects of food safety–associated risks on the international trade of food and related products. A total of 37 published studies retrieved using different search engines were included in this review. This review revealed that because of rapid population growth and rising food demand in developing nations, agricultural intensification is growing. It has been found that foodborne illnesses and associated discrepancies can impede the international trade of food commodities. Trade bans due to the fear of foodborne illnesses are growing. The consequences of foodborne diseases are multifaceted and include financial losses from trade restrictions, medical costs for prevention or control, resource depletion and a decline in food production. The overall effects are increased international trade tensions and livelihood vulnerability to poverty, notably for small‐scale livestock producers. Potential food contaminants include microbes, pesticides, pharmaceutical residues, heavy metals and fraudulent such as improper food processing, mislabelling, poor packaging, adulteration and substitution. Hence, countries are encouraged to harmonize the rights and duties set by the World Trade Organization under sanitary and phytosanitarys to maximize their advantages in global markets. Based on this evidence, we recommend that each country develop and integrate regulations that would ensure the safety of both domestic and international food production systems. Furthermore, the global community should either revise the current functioning food regulatory and monitoring body or establish a more genuine collaborative network.

## Graphical abstract

Food safety has become a pressing global issue due to growing health concerns. Contaminants can infiltrate the food supply chain, from agricultural production to processing and final consumption. Factors, such as improper agricultural inputs, microbial contamination in production and processing and fraudulent practices, are hindering international food trade. In response, extensive food safety regulations have been implemented. Yet, these regulatory measures may paradoxically create barriers to the global food market.

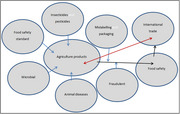

## 1 INTRODUCTION

Food safety is an essential public health concern worldwide. It is a crucial component of food security, which is receiving increasing global attention due to the growing number of widespread foodborne disasters (Unnevehr, 2015). Regulations related to food safety were first brought to the forefront after NASA required Pillsbury, a food processing company, to provide pathogen‐free food for a space mission in the early 1960s (Ibrahim, 2020; Weinroth et al., 2018).

Despite the clear link between population growth and increased demand for food, which has driven mandatory agricultural intensification in low‐ and middle‐income countries, there are growing concerns about food safety issues related to agricultural products, such as fruits, vegetables, meat, eggs and dairy products (Li et al., 2018). Discrepancies in food safety measures within the food supply chain of agricultural production can lead to the occurrence of foodborne diseases (FDs) (Odetokun et al., 2022). Even though global food trading surpassed US $1.5 trillion in 2017, the economic impact of FDs associated with financial losses was $50.8 billion per year for upper‐middle‐income countries (Afsana et al., 2022). About 600 million people (almost 1 in 10) suffer from foodborne illnesses globally, resulting in 420,000 deaths annually (Afsana et al., 2022).

Food safety concerns have become ubiquitous because of domestic food unavailability, market volatility and restrictions and emergency trade bans on the import and export of various food commodities (Salajegheh et al., 2022). Some dishonest food traders have found that unregulated markets give them an excellent opportunity to exploit consumers through unfair trade practices (Kenny, 1998). Consequently, numerous foodborne illnesses caused by the consumption of contaminated foods containing bacteria, viruses, mycotoxins, heavy metals, pesticides and pharmaceutical residues have become more prevalent (Kharel et al., 2022). Foodborne illnesses are a public health concern in both developed and developing countries, with the latter being known for frequent FDs (Song et al., 2014). Moreover, fraudulent food, such as adulteration, substitution, unhygienic processing and improper packaging, is a major concern (Song et al., 2014).

Unrestricted international trade of food could increase health risks, as food supply chains cross multiple national borders. The establishment of effective national food control standards is hence essential for protecting the health and safety of consumers. Thus, public health and safety rely on national food control systems, which enable countries to ensure that only safe and high‐quality food products enter the food market (World Health Organization [WHO]/Food and Agriculture Organization [FAO], 2003). The *Codex Alimentarius*, or ‘food code’, was established to set international standards to ensure the safety and quality of food and agricultural products and to create a playing field for international trade (Wieck & Jason H., 2021).

Nations must ensure the safety and quality of food before entering international trade, and imported food should comply with national standards. Ensuring these requirements requires strict implementation of food hygiene rules throughout the entire food production process, from preparation to preservation. All stages of food production must adhere to good agricultural practices (GAP) and the hazard analysis and critical control points system (Wieck & Jason H., 2021). The WHO recommends that countries implement and enforce risk‐based food control policies (Gizaw, 2019). The International Organization for Animal Health (OIE) also supports monitoring animal diseases that pose threats to public health (Acord & Walton, 2004). Typically, sanitary and phytosanitary (SPS) measures taken by smallholder farmers lead to border rejection (Li et al., 2018).

However, food safety standards can sometimes act as barriers to international trade. The effective implementation of food safety standards is limited by fragmented legislation, multiple jurisdictions and weaknesses in surveillance, monitoring and enforcement. According to Afsana et al. (2022), poorly managed food safety measures applied in developing countries lead to massive economic loss and waste. One of the greatest challenges faced by both developed and developing *Codex* member nations in adopting food safety standards is the economic implications of setting standards that are appropriate and reasonable (Wieck & Jason H., 2021). Moreover, the growth of food safety regulations has increased the complexity of trade policy discussions and attempts to expand agricultural trade. The costs required for firms, especially those in developing countries, to comply with these new food safety standards have led to concerns about a loss of competitiveness (Wieck & Jason H., 2021; Gizaw, 2019).

It has been practical for international food trade regulation to be violated for various reasons. These include the intentional disregard of regulations to maximize profits, inadequate infrastructure that fails to provide proper working conditions and massive and uncontrolled global demand for food. Persistent disease outbreaks associated with food contamination by pathogens and other substances, mislabelling and packaging, fraud and food additives are the most important threats to the international trade of food markets. The COVID‐19 pandemic, tariff disputes between China and the United States, and regional instability have severely affected international food trade and marketing (Salajegheh et al., 2022). In addition, growing and prevalent food standards and enforcements have threatened the international food market.

Therefore, the objective of this review was to identify the challenges associated with food safety in the international trade of food commodities. The possible questions for this review were as follows: (1) What are the main constraints on international trade? (2) What are the impacts of food safety measures on the global marketing of food commodities? (3) Which type of economic sector is severely affected by food safety–related issues? (4) Which class of country is most affected by food safety‐related measures?

## 2 MATERIALS AND METHODS

Previously published studies were retrieved using predetermined criteria that were built for the review. Initially, materials from the Scopus, Google Scholar and Web of Science databases were searched. The first 10 hits for Google Scholar results were retrieved. Titles and abstracts were assessed and screened in the online version. Afterwards, selected and retrieved materials were imported to Medley Reference Manager, and duplicates were removed automatically. The relevance of the materials was evaluated on the basis of the following criteria: The materials should be published in recent years with sufficient detail about food safety issues, the effects of international trade regulations and FDs in the global market and the effects of trade restrictions. After all, key information on the results, discussion and conclusions was extracted and analysed qualitatively. Materials published in languages other than the English language with no information about the implications of food safety on trade restrictions and public health impacts were excluded. For this review, 37 full‐text materials met the inclusion criteria. The included full‐text materials were research articles, short communications, review reports, regulatory papers, field inspections and research notes.

## 3 IMPACT OF FOOD SAFETY ON GLOBAL PUBLIC HEALTH AND THE ECONOMY

Food safety has mattered for public health since the 1960s when NASA commissioned a food processing company to make pathogen‐free meals at zero gravity in space (Weinroth et al., 2018). Food production and distribution systems, including open and global trade, are critical for meeting the dual goals of food security and global health in the first century (Adamchick & Perez, 2020).

Food safety rules and standards can hinder trade and substantially impact one's ability to access markets, especially in developing nations. To reduce food‐related dangers, safety regulatory regimes must first identify hazards to reduce food‐related dangers (Weinroth et al., 2018). Food safety issues, such as mislabelling, microbial contamination and chemical contamination, remain the most common causes of public health discrepancies. Developed nations have been preparing food safety standards and implementing food safety standards effectively (Gizaw, 2019). The development and adoption of food safety systems are very inconsistent among developing countries (Weinroth et al., 2018). Trade discrimination and restrictions imposed by developed countries on developing countries are also common phenomena that impede progress towards meeting international food safety standards (Li et al., 2018). Developing countries are struggling to harmonize international food safety standards (Song et al., 2014). Exports from developing countries to developed nations are often restricted by food safety restrictions imposed by developed nations. Outright bans are typically implemented as short‐term solutions when serious problems with food safety are discovered. According to the WHO, FDs can be caused by a wide range of factors, ranging from mild illnesses to chronic or life‐threatening illnesses (Song et al., 2014).

**FIGURE 1:**
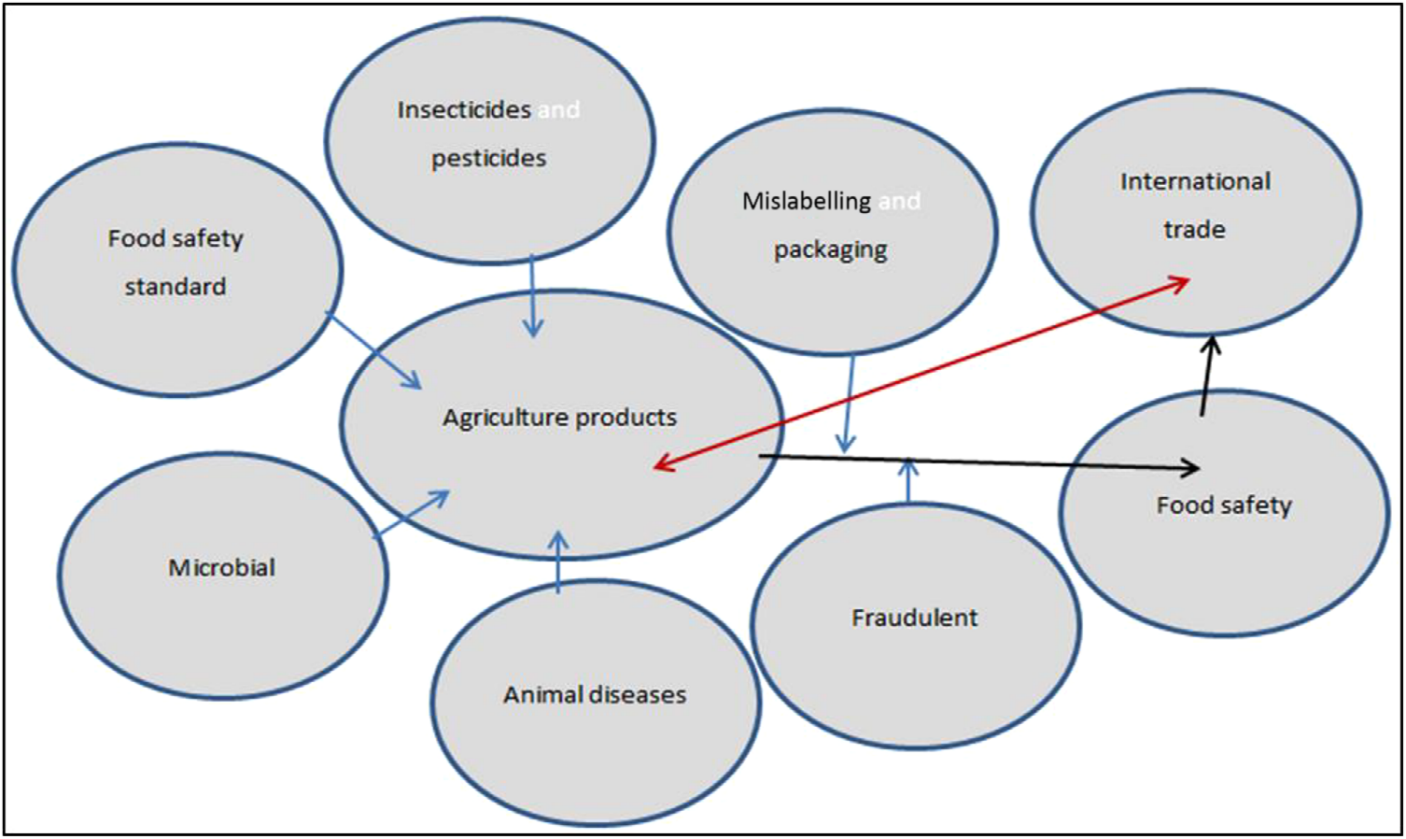
A schematic diagram depicting agricultural production, the trade chain, food safety risks and implications for international trade.

A wide range of biological and chemical agents, or hazards, cause foodborne diseases with varying degrees of severity, ranging from mild sickness to chronic or life‐threatening illness (Käferstein, 2003). A gap in knowledge, skills or attitudes regarding food handling in developing nations is the limiting factor in fulfilling international food safety standards. Microbial contamination, chemical contamination, food adulteration, misuse of food additives, mislabelling, outdated foods or foods past their use‐by dates are food safety‐related public health risks (Figure 1). Many substances belonging to the above categories have carcinogenic, mutagenic, teratogenic or toxic effects (Gizaw, 2019). Risks associated with food safety include cross‐contamination, a lack of consistency between food safety regulations and problems with developing in‐house resources. Moreover, consumers can be endangered by factors such as time delays, temperature abuse, inadequate hand washing and a lack of information (Pham et al., 2010). The increasing demand for safe food in developing countries entails meeting stringent food safety requirements. Food retailers and regulatory bodies impose food safety measures related to the production and handling of farm produce. For smallholders to remain competitive in such a system, institutional arrangements are necessary (Mwambi et al., 2020).

### 3.1 Bacteria

FDs can be caused by several factors, such as the virulence of microorganisms and the host immune system. Bacteria can cause life‐threatening infectious illnesses, such as meningitis or severe diarrhoea. Bacteria cause more than 52% of FDs (Islam, 2015). Several bacterial foodborne diseases result from biological contamination (Table 1). Certain bacterial contaminants have the potential to cause severe illnesses and fatalities (Ibrahim, 2020; Tavelli et al., 2022). Gizaw (2019) reported that local and international food marketing continue to have significant effects on the health of the public. Raw meat has the potential to encourage the growth and proliferation of microorganisms, which can act as a vector for the spread of disease (Odetokun et al., 2022). It has been reported that microbes can grow and survive on damaged tomatoes, increasing the risk of FD (Gemeda et al., 2022). The most significant diseases spread by unpasteurized, contaminated milk and milk products are tuberculosis and brucellosis (Gallo et al., 2020). The bacteria that pose the greatest risk to food safety are *Staphylococcus*, *Streptococcus*, *Salmonella*, *Listeria*, *Campylobacter*, *Bacillus*, *Clostridium*, *Brucella*, *Escherichia coli*, *Klebsiella* and others (Gallo et al., 2020).

**TABLE 1:** Major biological, chemical and physiological contaminates of food.

Bacterial	Fungal/parasitic	Pesticides/insecticides/poison	Heavy metals	Viral
*Campylobacter*	*Cryptosporidium*	Gambier discus		
*Shigella* *Yersinia* *Vibrio vulnificus* *Escherichia coli, Listeria monocytogenes, Staphylococcus aureus, Clostridium botulinum* *Salmonella*	*Protoperidinium crassipes* *Lamblia aspergillus* *Aspergillus flavus* *Verrucosum Protoceratium*, *Reticulatum Lingulodinium, Toxin Fusarium Toxin, Fusarium Poae, Ascaria, Trematodes* *Cyclospora giardia*	Toxicus Brevetoxin shellfish Poisoning Ciguatoxins Azaspiracid Aflatoxin B1, B2, G1 and G2, M1, DDT, microgram	Cadmium Mercury Arsenic Pb	*Rotavirus* *Norovirus* *Human astrovirus* *Sapovirus* *Adenovirus*

### 3.2 Parasites

Ascariasis is one of the most common parasitic diseases, with an estimated one billion cases annually worldwide (Gallo et al., 2020). Trematode infections have been reported in more than 40 million people, primarily in Asia, Africa and Latin America. Globally, 10% of the population is at risk from these parasites, which are transmitted through the consumption of raw or inadequately cooked freshwater fish, shellfish or aquatic plants (Käferstein, 2003). Many parasites can be eliminated by cooking or by very low temperatures, whereas others can live in the human body, sometimes for decades, causing serious damage to human health. *Giardia lamblia*, *Cryptosporidium* sp. and *Entamoeba histolytica* are important pathogenic intestinal parasites and are among the leading causes of diarrhoeal illness in humans worldwide (Di Genova & Tonelli, 2016). *Amblyomma variegatum* is a tropical bond tick that causes heavy economic losses in Africa and Caribbean islands. The ability of ticks to reach the American continent can result in economic damage of 1 billion US dollars (Domenech et al., 2006).

### 3.3 Viruses

FDs can be transmitted between species, including humans, and some viruses become dangerous in other species. Hepatitis A, the most common viral disease transmitted by food, is caused by the consumption of contaminated water, foods that are raw or poorly cooked, or vegetables washed with contaminated water. In most cases, the seafood and transmitter virus responsible for this pathology is *hepatitis A virus*, a picornavirus that is currently considered the prototype of hepatovirus (Gallo et al., 2020).

Pigs can be used as mixing vessels for *Avian influenza* (Focker et al., 2022). *Rotavirus*, *norovirus*, *spovirus* and *adenovirus* are among those that cause severe disease (Gallo et al., 2020). Two *coronaviruses*, *Porcine epidemic diarrhea virus* (PEDv) and *Porcine Delta Coronavirus*, caused extremely contagious swine illnesses and devastated North America in 2013. PEDv accounted for 10% of the deaths of US domestic pigs, with losses of $1 billion in 1 year. *African swine fever*, which has ravaged in Asia and Europe since 2007, has wiped out the domestic pig population in China (Adamchick & Perez, 2020). In 2007, during the outbreak of *Rift Valley Fever* in Kenya, the outbreak‐related financial loss was estimated at US $32 million (Smith et al., 2017). Infections by pathogens, such as foot‐and‐mouth disease, have resulted in billions of dollars in losses to producers (Acord & Walton, 2004). In 2015 and 2003, China and South Korea lost $2.6 billion and $17 billion, respectively, due to the MERS outbreak. In 2009, Canada, the US, Mexico and the UK lost $31 million, $250 million and $20 million, respectively, from H1N1 outbreaks (Rahimi et al., 2022).

Transboundary animal diseases (TADs) pose social and economic costs and risks to countries, their neighbours or their trading partners. The varying impact of TADs among stakeholders and the threat of trade partner countries complicated appropriate control measures. For all livestock producers, the threat of TADs increases the risk of lost production and impacts livelihoods, increasing vulnerability to poverty, particularly for small‐scale producers (Domenech et al., 2006). Further food safety issues are related to losses in consumer confidence.

### 3.4 Pesticides

Pesticides play an important role in controlling crop diseases and insect pests to improve crop quality. Preventative measures are being expanded to include fruits and vegetables in the United States after outbreaks of foodborne illnesses caused by microbial contamination. Any germs that may have developed in the meal are usually killed by heating them before consumption, and cooking is a good way to kill them (Li et al., 2018). Because most pesticides are difficult to degrade and toxic, residual pesticides from food and the environment can cause slow poisoning after they enter the human body. Some pesticides can even directly inhibit the activity of enzymes (Tang et al., 2022). Foods contaminated with pesticides or other chemicals are an important cause of cancer (Nguyen‐Viet et al., 2017). Organic reactions are chemical reactions caused by the use of pesticides and herbicides in the cultivation of food. FDs can be caused by exposure to agricultural substances, such as pesticides, insecticides, herbicides, rodenticides, fertilizers, cleaning residues and naturally occurring toxins (Afsana et al., 2022). The three most commonly implicated pesticides are diazinon, malathion and organophosphate (Nguyen‐Viet et al., 2017). Agrochemical agents, such as fungicides, pesticides or herbicides, increase the levels of plant toxins and mycotoxins in food (Jeddi et al., 2022).

Pesticides are toxic not only to pests but also to humans, animals and the environment. Chronic health risks may also occur, including cancer; diseases of the heart, nervous system, kidney and liver; and disruption of endocrine function. The levels of pesticide residues should be controlled in food. The use of pesticides for the control of mycotoxigenic fungi is controversial. Antibiotics are used to maintain animal health and prevent bacteria from entering the food supply (Altomare et al., 2021). The application of pesticides and fertilizers by vegetable growers should be regulated to ensure correct use and the obligation not to exceed the maximum residue limits. Government extension programs should be targeted to promote GAP in vegetable production (Kharel et al., 2022). In addition, excessive pesticide residues pose a great threat to food safety (Wang et al., 2022). The uncontrolled use of pesticides in fruits, vegetables and cereals is worrying (Oskarsson, 2012). Residues of pesticides remain in the soil, and the amount of pesticide in fruits is reportedly low (Focker et al., 2022). The attempt to increase yields from limited lands was achieved by chemical pesticides or by planting high‐yield crops (Islam, 2015). According to some experts, pesticides may cause endocrine disruption. Thus, using good practices, such as proper farmyard manure application and biopesticides, is imperative for sustainable vegetable production (Kharel et al., 2022).

### 3.5 Fraudulent

Excessive use of food additives or the excessive consumption of exotic foods can lead to an increase in the risk of developing an adverse reaction (Weinroth et al., 2018). Obligatory and profit‐driven practices, such as food fraud and food adulteration, have also exacerbated food safety issues (Odetokun et al., 2022). Foods in the international market may be fraudulent, as different parties, such as manufacturers, packers, distributors and others, are involved in the chain of distribution. Fraudulent food practices such as adulteration, mislabelling and selling of spoiled or expired foods also cause microbial contamination (Gizaw, 2019). Developed and developing countries still struggle with the uniform regulatory implementation of food safety standards. Developed countries all have the core components of reducing foodborne illness, such as traceability, sustainability, food fraud or food defence. Moreover, developing countries do not have the same level of standardization or consistency in their food safety systems (Weinroth et al., 2018). Hashaam et al. (2021) reported that expired food is the leading cause of foodborne illness in more than half of Pakistan's population.

### 3.6 Packaging and mislabelling

About 30% of the annual world's foods are wasted as improperly produced and packaged products. Food packaging is critical for safeguarding food quality and reducing food waste, according to the World Food Programme (Wang et al., 2022). Packaging can reduce food waste by extending shelf life, preventing microbial spoilage and reducing the fuel consumption used to transport raw materials (Matthews et al., 2021). There is a critical need for improved packaging systems, refrigerated transport and cold chains for perishable produce for better product quality and food safety (Gemeda et al., 2022). All plastic packaging in the EU should be either reusable or efficiently recycled by 2030 (Focker et al., 2022). However, recycling plastics for the reuse of food packaging can pose a risk, as they are non replenishable and do not decompose in the same way as paper or/span>metal. Food packaging materials have the potential to be contaminated, compromising the public health of the community (Gizaw, 2019).

Infectious agents can disseminate from original processing and packaging locations thousands of kilometres away because of the globalization of food production, manufacturing and marketing (Domenech et al., 2006). More recycled materials are being used in food packaging as part of efforts to produce food in a more sustainable manner, which leads to more contamination by dangerous chemicals (Jeddi et al., 2022). Waste and inappropriate packing issues might also emerge during transportation (Zhong et al., 2022). Environmental hazards, including foreign bodies and physical hazards, could have the potential to cause serious illness and injuries. In addition, plastic waste from nano‐ and microplastics can enter or re‐enter the food chain (Jeddi et al., 2022). A review of food businesses in developed countries showed that mislabelling is the most common problem concerning food safety (Gizaw, 2019). Food packaging in developing countries is often inadequate due to low domestic demand and low investment. Between harvest and processing, 40% of food in developing countries can be lost (Matthews et al., 2021). Biosensors could play a significant role in the detection of contaminants in packed food (Wang et al., 2022).

### 3.7 Food additives

Substances generally recognized as safe can be used as food additives; however, misuse (maximum allowable concentration), the use of nonpermitted substances and the blending of permitted and nonpermitted substances together cause health hazards (Gizaw, 2019). Food additive items can be added to produce a sweet taste, smell, impart colours, increase shelf life (preservation), act as antioxidants, solidify the product (increase viscosity), maintain acidity, dissolve, dilute or melt products and prevent the caking of the product to the containers and packing gases (Gallo et al., 2020). Food additives and the unregulated use of antibiotics have contributed to increased incidence of non‐communicable diseases such as cancer, diabetes and obesity.

### 3.8 Drug residues

One of the most important breakthroughs in medical history is the discovery of antimicrobial agents. Antibiotics are widely used in animal production for therapeutic, prophylactic and growth enhancer purposes (Alemu et al., 2020). However, excessive reliance on these substances has led to the development of antimicrobial resistance and negative health effects, such as allergies, which have emerged as a public health concern worldwide [(El‐zamkan & Mohamed, 2021; Ndirangu et al., 2015) and (Tsugami et al., 2022)]. Antibiotic residue can be present in animal‐origin foods such as eggs, meats and milk (Novaes et al., 2017). In addition, more than half of the drug doses given to animals are excreted unchanged via urine and faeces, which can in turn affect the microbial community of the soils and crops to which they are applied. Manure may be heat‐treated but inefficient at removing all pathogens (Focker et al., 2022). Microbiological hazards are barriers to the reuse of human and animal faeces, and antimicrobial resistance could lead to an inability to manage outbreaks and treat diseases with antibiotics 5. The inefficient use of antimicrobials in animal production and aquaculture in many areas of the world has drawbacks, such as negative effects on the environment and human health (Jeddi et al., 2022). Antibiotic resistance in bacteria poses a major global health threat by rendering antibiotics ineffective for treating infections, according to the WHO and the US National Institute of Antibiotic Respiratory Medicine, which aim to protect against antibiotic‐resistant superbugs (Tavelli et al., 2022). Antimicrobial resistance is a major global public health concern and a food safety issue (FAO et al., 2022).

### 3.9 Chemicals and heavy metals

Toxic metals are substances that have no physiological or biochemical functions and are not commonly present in the human body (Islam, 2015). Foodborne illnesses could result when those substances contaminate food or water during the manufacturing process, distribution or retailing or when the food enters the body. Chemicals and heavy metals in contaminated food can lead to poisoning and long‐term diseases such as cancer (Ibrahim, 2020). Heavy metals, such as arsenic, bromine and iodine (Jeddi et al., 2022), cadmium, nickel, lead, copper, zinc, iron, mercury and manganese, are chemical food contaminants (Gizaw, 2019; Islam, 2015).

### 3.10 Genetically modified organism

Many countries are reluctant to import genetically modified or gene‐edited foodstuffs for regional consumption (Anderson, 2022), which in turn hampers the global trade of food and related commodities. Many GMO crops are used to make food ingredients, such as cornstarch, corn syrup, corn oil, soybean oil, canola oil or granulated sugar (Food and Drug Administration [FDA], 1995). Allergic responses, cancer, diabetes, obesity and undesired side effects, such as toxicity, organ damage or gene transfer, are major concerns regarding the use of genetically modified organisms. A study conducted in China indicated that genetically modified food items are the main factor affecting consumers’ decisions regarding ready‐to‐eat meals (Shao et al., 2021). A study in Kenya and Australia revealed that consumers are reluctant to purchase genetically modified foods. Genetically modified crops may contain allergenic compounds (inserted novel genes), resulting in antibiotic‐resistant bacterial strains (Gizaw, 2019). Overall, this fear is driven by the belief that genetically modified foods (bioengineered food), as sources of the aforementioned concerns, could hinder the global food trade.

## 4 GLOBAL TRADE DISPUTE MITIGATION

The World Trade Organization (WTO) outlined a dispute settlement method if a country violently imposed restrictive measures on other nations (Adamchick & Perez, 2020). The WTO negotiates to minimize trade wars via its most favoured nation and reciprocity articles and the dispute settlement mechanism. Transparency and inclusiveness in policy debates, compensation for losers and long transition periods will need to be among the strategies adopted by politicians and international negotiators (Anderson, 2022). OIE standards are recognized by the WTO as reference international sanitary rules. OIE has a network of laboratories and collaborating centres that provide scientific and technical support and expert advice on topics linked to disease surveillance and control. The increasing importance of trade and expanded access to world markets by developing countries have also received attention from the WTO (Adamchick & Perez, 2020).

The SPS Agreement of the WTO gives permission that countries take legitimate measures to protect the public's life and health. The agreement requires that risks be kept at an acceptable level. WTO members are asked to accept the food safety measures of other members if they impose an equivalent level of protection. Food safety regulations and standards are increasingly influencing the ability of developing countries to access markets for agricultural and food products, particularly in industrialized countries. Most of the effects of food safety requirements on trade stem from government regulation, but they can also come from industry and other sources (Song et al., 2014) For trade in animals and animal products, countries must also adhere to the standards, guidelines and recommendations established by the OIE (Thiermann, 2005).

The Codex Alimentarius Commission (CAC) plays a significant role in influencing national food safety regulations by setting international food safety standards. These standards are used as guidance for national food safety regulation and can be referenced in WTO trade disputes (Wieck & Jason H., 2021). The CAC food standards, guidelines and codes of practice contribute to the safety, quality and fairness of this international food trade. Consumers can trust the safety and quality of the food products they buy, and importers can trust that the food they order will be prepared according to their specifications. Public concerns about food safety issues often place the Codex at the centre of global debates. Veterinary drugs, pesticides, food additives and contaminants are discussed in Codex meetings. Codex standards are based on sound science provided by independent international risk assessment or consultation bodies organized by the FAO and WHO (FAO et al., 2022).

The international trade of food of animal origin (particularly meat and dairy) is restricted by SPS measures related to human and animal health, but SPS is widely traded and consumed in developing countries. The fundamental difference between domestic markets in developing countries and those in developed nations is that the latter are more open and consumer‐oriented. Developed countries need confidence that food safety standards in exporting countries will provide an equivalent level of safety to what they seek from their domestic food safety systems (Li et al., 2018). However, the food safety regulatory body (WTO/SPS) does not receive trust from developing nations, which benefits wealthy nations. Restrictions on mobility and national and international trade have disrupted animal markets and access to consumers (Rahimi et al., 2022). Both developed and developing nations must strike a balance between reducing human health risks and securing economic benefits of food safety standards vary between developed and developing countries in production and trade when setting food safety standards (Wieck & Jason H., 2021).

## 5 CONCLUSION AND RECOMMENDATIONS

Food safety concerns are becoming increasingly common worldwide. Any inconsistency or problem in the food supply chain, from production to storage and preservation, can lead to foodborne illnesses. The main contaminants found in food include microorganisms, pesticides, drug residues and heavy metals. The economic impact and public health risks associated with FDs are growing concerns, the theory and practice. As a result, food safety standards have the potential to become barriers to international trade. Developed countries have a greater ability to both develop and implement effective food safety standards. In contrast, the food safety measures used in developing countries often result in substantial economic losses and disruptions. GAP and hazard analysis are crucial techniques for ensuring food safety. Thus, I recommend that there should be an agreed‐upon regulation worldwide to ensure safe food production. In addition, the present regulatory system should be revised or assembled in a new, more genuine and suitable format.

## AUTHOR CONTRIBUTIONS

**Abebe Tibebu**: Data curation; formal analysis; methodology; supervision; writing — original draft; writing — review and editing. **Habtamu Tamrat and Adane Bahiru**: Methodology; supervision; writing—original draft; and writing — review and editing.

## CONFLICT OF INTEREST STATEMENT

All of the authors have stated that there are no conflicts of interest.

## FUNDING INFORMATION

None.

### ETHICS STATEMENT

The authors confirm that the ethical policies of the journal, as noted on the journal’s author guidelines page, have been adhered to. No ethical approval was required as this is a review article with no original research data.

### PEER REVIEW

The peer review history for this article is available at https://publons.com/publon/10.1002/vms3.1585.

## Data Availability

No data are available.
